# How to use cardiac troponin in non-cardiac surgery

**DOI:** 10.1093/ehjacc/zuad057

**Published:** 2023-05-31

**Authors:** Christian Puelacher, Nicholas L Mills, Christian Mueller

**Affiliations:** Cardiovascular Research Institute Basel (CRIB) and Department of Cardiology, University Hospital Basel, University of Basel, Petersgraben 4, 4031 Basel, Switzerland; BHF Centre for Cardiovascular Science, University of Edinburgh, Edinburgh, UK; Usher Institute, University of Edinburgh, Edinburgh, UK; Cardiovascular Research Institute Basel (CRIB) and Department of Cardiology, University Hospital Basel, University of Basel, Petersgraben 4, 4031 Basel, Switzerland

Over 300 million surgical procedures are performed annually, with demand for surgery likely to increase in the coming years with an aging population.^1^ Although medicine has made many advances, major non-cardiac surgical procedures still carry a significant risk of cardiac complications and death. The 2022 ESC ‘Guidelines on cardiovascular assessment and management of patients undergoing non-cardiac surgery’ have endorsed use of cardiac troponin (cTn) T and I, to help improve perioperative outcomes.^2^

The guideline recommends the use of cTn in patients at higher risk in two situations: firstly, for preoperative risk stratification, and secondly, for the detection of perioperative myocardial infarction/injury (PMI), using an active surveillance strategy involving serial cTn measurements, starting preoperatively and continuing on postoperative days 1 and 2.^2^

Perioperative myocardial infarction/injury, defined as an acute increase in cTn concentration compared to preoperative concentration, is a pathophysiological hallmark of nearly all perioperative cardiovascular complications and can easily and inexpensively be detected by serial measurements.^2–5^ First studies estimated that PMI is much more common than expected (16% in high-risk patients)^3^ and a major contributor to 30-day mortality after non-cardiac surgery within 30 days.^6^ Most patients with PMI do not exhibit typical ischaemic symptoms,^3,6^ making the asymptomatic presentation insidious. In fact, asymptomatic PMI has been shown to have a similar impact on 30-day mortality as symptomatic PMI (10.4% vs. 8.7%, *P* = 0.68).^3^

The term PMI includes ‘infarction/injury’ to highlight that at time of detection via acute cTn increase, the predominate aetiology of cardiomyocyte injury remains to be determined. The causes of PMI vary widely, from type 1 myocardial infarction due to thrombotic plaque rupture, to type 2 myocardial infarction caused by demand–supply mismatch due to bleeding, hypotension, or tachyarrhythmias, to acute heart failure and severe sepsis causing myocardial injury.^4^

Due to this heterogeneity, detailed clinical evaluation of patients with PMI is paramount. While all aetiologies are associated with worse outcomes, stark differences exist according to presumed aetiology, with 1-year mortality reaching 49% in PMI of acute heart failure type, 40% in tachyarrhythmia type, and 39% in primarily non-cardiac cause PMI.^5^ Beside the difference in associated outcome, the time from diagnosis of PMI to further complications is also markedly shorter in PMI of the acute heart failure (median 3 days) compared with those with type 2 myocardial infarction or tachyarrhythmia (median 13 days). Hence, the window of opportunity to improve outcome is dependent of aetiology as well. In most cases, the first investigation following clinical evaluation will be a transthoracic echocardiography, ideally performed on the day of PMI detection.^5^

**Figure 1:**
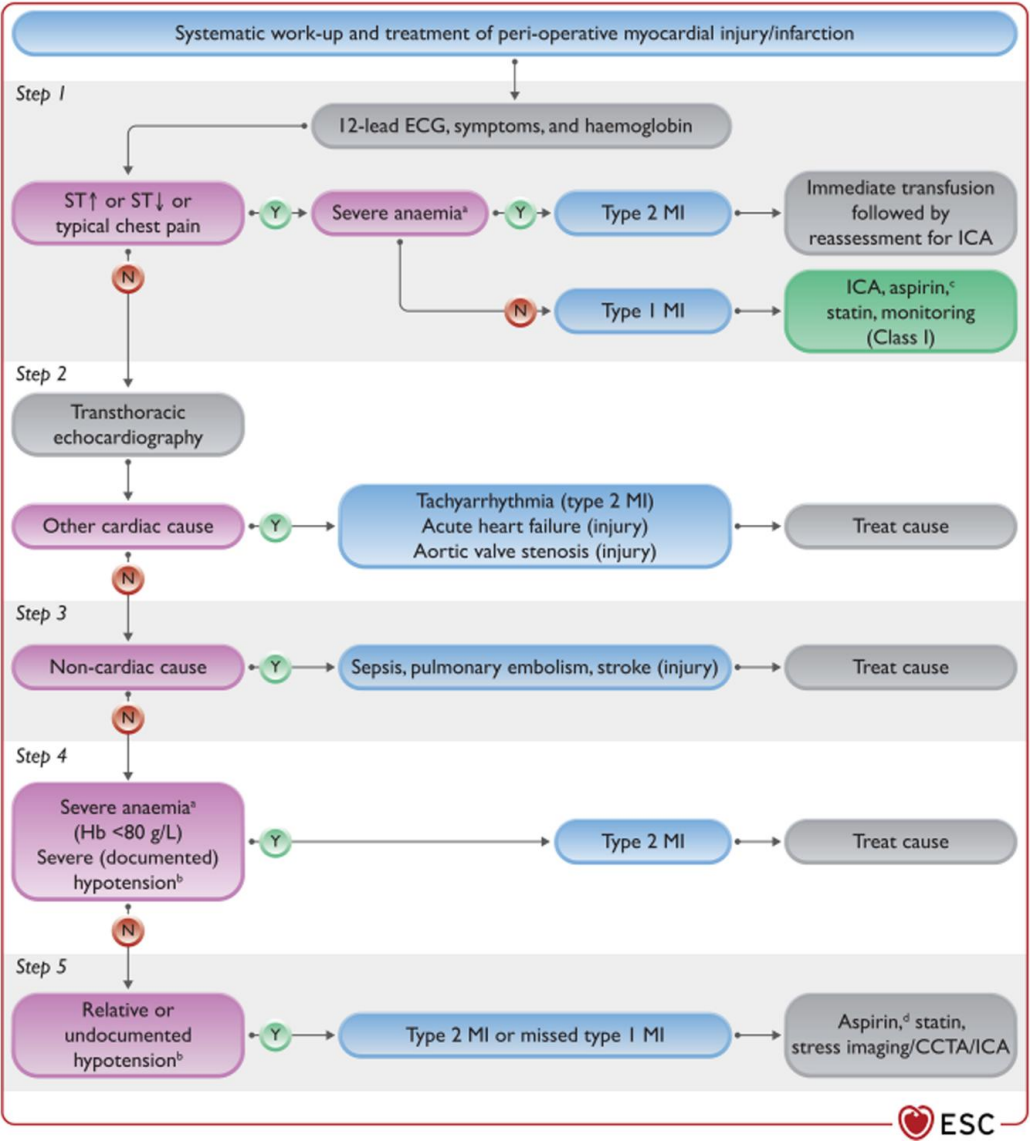
(adapted from ESC^2^) Diagnostic and management algorithm for perioperative myocardial infarction/injury following detection via active surveillance. Different perioperative cardiovascular complications will result in perioperative myocardial infarction/injury and determining aetiology guides optimal therapy. CCTA, coronary computed tomography angiography; ECG, electrocardiogram; Hb, haemoglobin; ICA, invasive coronary angiography; MI, myocardial infarction; ST, ST-segment. ^a^Or active bleeding. ^b^Or other type-2 MI trigger such as hypoxaemia, tachycardia, and hypertension. ^c^Dual antiplatelet therapy after coronary stenting. ^d^Possibly in combination with dabigatran 110 mg b.i.d.

The current guidelines proposes a diagnostic and management algorithm (*Figure 1*) focusing on determining the underlying aetiology and implementing aetiology-specific treatment strategies from the non-surgical setting, with the aim of improving perioperative outcomes by interdisciplinary collaboration following non-cardiac surgery.^2^
